# Functional divergence of the rapidly evolving miR-513 subfamily in primates

**DOI:** 10.1186/1471-2148-13-255

**Published:** 2013-11-19

**Authors:** Zhenghua Sun, Yanfeng Zhang, Rui Zhang, Xuebin Qi, Bing Su

**Affiliations:** 1 Yunnan Key Laboratory of Primate Biomedical Research, Kunming 650217, China; 2 Kunming Institute of Zoology, State Key Laboratory of Genetic Resources and Evolution, Chinese Academy of Sciences, Kunming 650223, China

**Keywords:** miR-513, Primate-specific, Rapid evolution, Testis, DR1

## Abstract

**Background:**

The miR-513 subfamily belongs to an X-linked primate-specific miR506-514 cluster. Across primate species, there have been several duplication events and different species each possess a variety of miR-513 copies, indicating it underwent rapid evolution. Evidence suggests that this subfamily is preferentially expressed in the testis, but otherwise, to date, the evolutionary history and functional significance of this miRNA subfamily has remained largely unexplored.

**Results:**

We analyzed the evolutionary pattern of gene duplications and their functional consequence for the miR-513 subfamily in primates. Sequence comparisons showed that the duplicated copies of miR-513 were derived from transposable element (MER91C). Moreover, duplication events of the miR-513 subfamily seem to have occurred independently in *Platyrrhini* (New World monkeys) and *Catarrhini* (Old World monkeys, apes and humans) after they diverged. Different copies of the miR-513 subfamily (miR-513a/b/c) have different seed sequences, due to after-duplication sequence divergences, which eventually led to functional divergences. The results of functional assays indicated that miR-513b could inhibit the expression of its target gene, the down-regulator of transcription 1 (DR1) at both the mRNA and protein levels. In the developing testis of rhesus macaques, we observed a temporal coupling of expression levels between miR-513b and DR1, suggesting that miR-513b could affect male sexual maturation by negatively regulating the development-stage related functioning of DR1.

**Conclusions:**

The miR-513 subfamily underwent multiple independent gene duplications among five different lineages of primates. The after-duplication sequence divergences among the different copies of miR-513 led to functional divergence of these copies in primates.

## Background

MicroRNAs (miRNAs) are small (~22nt) endogenous non-coding RNAs that function as post-transcriptional gene regulators of a variety of biological processes [1-3]. Most miRNAs discovered at the early stage are highly conserved among species, indicating strong functional constraints on miRNA evolution. However, an increasing number of rapidly evolving miRNAs have been discovered and experimentally verified [4]. In an evolutionary study, Bentwich *et al.* reported two miRNA clusters in primates with more copies than those present in rodents, suggesting miRNA gene expansion during primate evolution. One of the two clusters, the miR-506-514 cluster, is located on the X chromosome and is preferentially expressed in testis [5]. Zhang *et al.* reported that this cluster has undergone rapid evolution in primates, with frequent gene duplications and nucleotide substitutions. In rhesus macaques, for example, expression analysis revealed a strong correlation between miRNA expression changes and male sexual maturation [6]. Unfortunately, to date, the functional significance of these duplicated miRNAs has not been experimentally established.

In the miR-506-514 cluster, the miR-513 subfamily has the greatest diversity in terms of both copy number and sequence variations [6]. There are also between-copy sequence substitutions in the mature miRNAs, especially in the seed region, which are critical for gene targeting. These observations collectively suggest that members of the miR-513 subfamily may play different roles in gene regulation.

In the most general sense, studying non-conserved miRNA may help to clarify how miRNAs originate and evolve, and likewise contribute to functional divergence among species. In the present study, we investigated the molecular mechanisms underlying the frequently observed gene duplications and sequence divergences in the miR-513 subfamily, and evaluated the impact of these sequence substitutions on target gene regulation and functional diversification.

## Results

### MiR-513 is a rapidly evolving miRNA subfamily in primates

**Figure 1:**
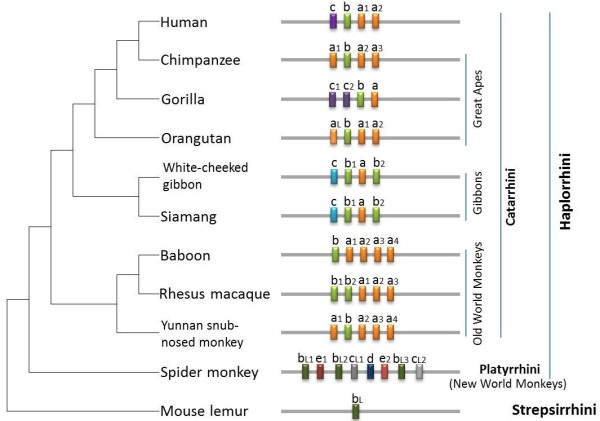
**Distribution of the miR-513 subfamily in primates.** Physical locations of each miRNA is indicated in the bar scheme. Gene copies of the miR-513 subfamily are named from “a” to “e” based on their sequences in miRBase. Different colors were used to show the classification of the mature miRNAs, and copies with the same color have the same mature miRNA sequences. If the mature miRNA sequence is different while the seed region is the same, the miR-513x is named as miR-513xL. The species tree was based on the NCBI taxonomy tree.

The miR-513 subfamily belongs to the miR-506-514 cluster located on the X chromosome. According to the miR-513 annotation in miRBase (http://www.mirbase.org) and remapping the miR-513 precursor sequences to primate species’ genomes that are sequenced (Additional file 1: Table S1), aside from one orthologous copy in the rat genome (rno-miR-3585), miR-513 exists only in primates. Among the primate lineages, the mouse lemur (a prosimian monkey in *Strepsirrhini*) possesses only one copy, where there are multiple gene copies in *Haplorrhini* (New World monkeys, Old World monkeys, apes and humans) (Figure 1)*.* For example, the spider monkey (a New World monkey) possesses eight copies, and Old World monkeys possess five copies, while gibbons, great apes and humans all possess four copies. These copies are named from “a” to “e”, based on their sequences in miRBase. Interestingly, after alignment with known repeat sequences, the miR-513 copies seem likely to have been derived from MER91C (Additional file 1: Table S2), a DNA transposable element [7]. All miR-513 copies are co-localized with MER91C and their sequences are similar (>50 identify; Additional file 1: Table S2). Other genes of the miR-506-514 cluster, however, are not similar with MER91C, suggesting that the mechanism behind gene duplications in the miR-513 subfamily is different from the others.

**Figure 2:**
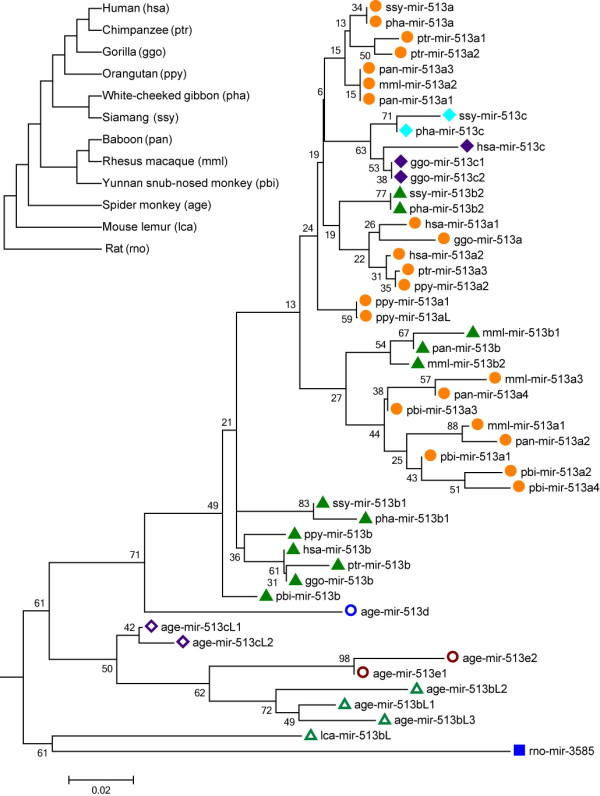
**Phylogenetic tree of the miR-513 subfamily.** The phylogenetic tree was reconstructed using the Neighbor-Joining method in MEGA5 and evaluated with 1,000 bootstrap replications (bootstrap values are labeled along the branches). Copies marked with the same colors and shapes have the same or similar mature miRNA sequences. The left top tree shows the corresponding species relationships among primates and the abbreviations for miRNA nomenclature. The scale bar reflects 0.02-nt substitution per site.

To further investigate the origin of duplication events among gene copies of miR-513 in primates, we reconstructed the phylogenetic tree using the miR-513 precursor sequences from the aforementioned primate species. Both neighbor-joining (NJ) and maximum likelihood (ML) methods were used for tree construction and the topologies of the trees by these two methods are highly similar (Figure 2 and Additional file 1: Figure S1). We also used the AnGST program to produce a chimeric gene tree (Additional file 1: Figure S2), and the tree topology remains the same. Interestingly, most copies from the spider monkey cluster together, and the gene order of the eight miR-513 copies suggests a tandem duplication of a fragment containing three copies (e1-bL2-cL1/e2-bL3-cL2). The age-mir-513d is not clustered together. By contrast, in *Catarrhini* (Old World monkeys, lesser apes, great apes and humans) the clustering of miR-513 copies is not in agreement with the accepted species phylogeny. Instead, copies with same or similar mature miRNA sequences cluster together (Figure 2), indicating ancient duplications of miR-513 in the common ancestor of *Catarrhini* after the divergence from *Platyrrhini* (New World monkeys), but before the Old World species split. By using AnGST and Notung, we also reconciled the gene trees with the species tree to infer duplication events along the branches, among which many independent gene duplications occurred in different primate lineages (Additional file 1: Figure S2 and Additional file 1: Figure S3).

**Figure 3:**
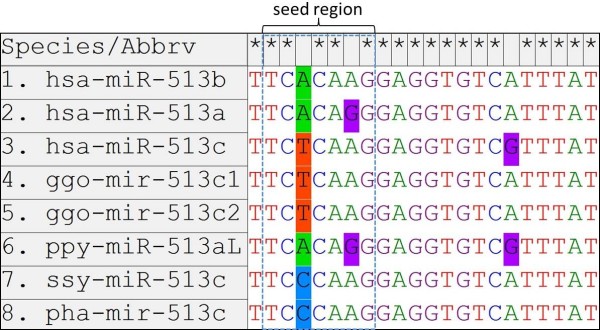
**Sequence alignment of mature miR-513c copies in gibbons, great apes and humans.** hsa-miR-513a and hsa-miR-513b were used as reference sequences. The “seed region” indicates the nucleotides 2–8 at the 5′ end of the mature sequences.

As reflected in the phylogenetic tree and sequence alignment of mature miR-513 sequences (Figure 2 and Additional file 1: Figure S4), miR-513b is probably the ancestral copy in *Catarrhini*, while miR-513a and miR-513c are derived copies generated by duplications. This inference was confirmed by the seed sequence comparison showing the same seed region in miR-513b with its orthologous copy in rats and an ancestral copy in the mouse lemur while both miR-513a and miR-513c have a single-base difference from miR-513b in the seed region (Additional file 1: Figure S4). Additionally, in *Catarrhini*, the formation of miR-513c was after the Old World species split and exists in apes and humans. Sequence alignment suggests that miR-513c emerged independently in gibbons, and there are varied copies in great apes and humans (Figure 3). For all three known great apes, there are two miR-513c copies in gorillas which were derived from miR-513b and lost in chimpanzees. The single miR-513aL copy in orangutans was derived from miR-513a according to the phylogenetic tree and sequence alignment. Notably, all miR-513aL/c copies have a single-base substitution (one of two sites) from their original sequence in gibbons and great apes, while the human miR-513c owns both substitutions (Figure 3). Likewise, sequence divergences among the miR-513 copies have occurred, consistent with the previously proposed rapid evolution of the miR-506-514 cluster.

### Functional divergence among gene copies of the miR-513 subfamily

**Figure 4:**
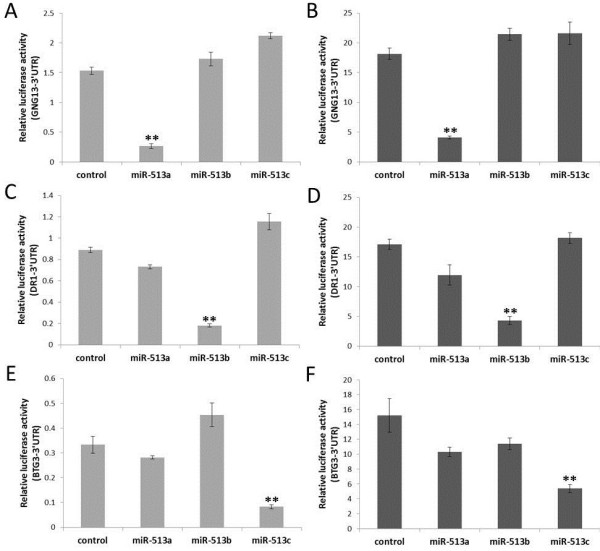
**Identification of miR-513 binding sites by luciferase assays.** The Y-axis represents the ratio of Renilla luciferase (with candidate target gene’s 3′ UTR) activity to firefly luciferase activity after treated with negative control or miR-513 mimics. **(A)** and **(B)** show miR-513a’s specific targeting of predicted sites in the 3′UTR of GNG13. **(C)** and **(D)** indicate miR-513b’s specific targeting of the 3′UTR of DR1. **(E)** and **(F)** display miR-513c’sspecific targeting of the 3′UTR of BTG3. **A**, **C**, **E** are results in HEK293T cells, while **B**, **D**, **F** are results in HeLa cells. Error bars represent standard deviations (n = 3). ** *P* < 0.01 (two-tailed student’s t-test).

Since the seed region of miRNA is critical for gene targeting, the between-copy nucleotide differences may potentially lead to functional divergence among gene copies of the miR-513 subfamily. To test this hypothesis, we searched potential target genes by TargetScans, a widely used method with low false positive rate. We then selected eight predicted target genes for luciferase assay testing. These target genes are GNG13 (Gene ID: 51764), DR1 (Gene ID: 1810), YBX2 (Gene ID: 51087), CTNS (Gene ID: 1497), BTG3 (Gene ID: 10950), NIPAL4 (Gene ID: 348938), IL13RA1 (Gene ID: 3597) and PI4K2B (Gene ID: 55300). All target sites in these genes are the same among different primate species, and we therefore used human genes for experimental validation. The change of secondary structure due to the length of 3′ UTR has been known to potentially affect the binding between miRNAs and their target sites. To avoid this, we cloned the full-length 3′ UTRs of the candidate genes for use in a luciferase assay. We used both HeLa and HEK293T cell lines to exclude the influence of different cell environments. The results showed that miR-513a, miR-513b and miR-513c are all capable of targeting their predicted sites in the 3′UTRs of GNG13, DR1 and BTG3 respectively (Figure 4 and Additional file 1: Figure S5), while the remaining five genes showed no evidence of significant regulation (Additional file 1: Figure S6). Notably, the miRNA-dependent inhibition of luciferase expression is specific for each member of the miR-513 subfamily (Figure 4), suggesting that different copies (miR-513a/b/c) with diverged seed sequences developed functional divergence by targeting different genes.

### Expression of GNG13, DR1 and BTG3 in testis of rhesus macaques

**Figure 5:**
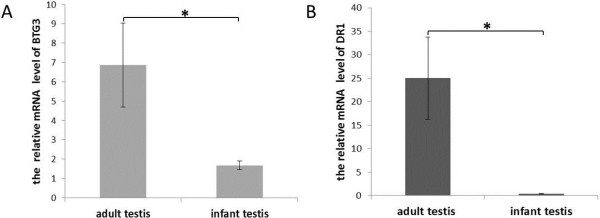
**Expression analysis of BTG3 and DR1 in testis of infant and adult rhesus macaques. (A)** mRNA levels of BTG3 in the testis of infant and adult rhesus macaques determined by real-time qPCR. **(B)** mRNA levels of DR1 in the testis of infant and adult rhesus macaques determined by real-time qPCR. β-actin was used as the internal control gene. Three infant and three adult samples were used. Error bar represents standard error (n = 3). *P < 0.05 (two-tailed student’s t-test).

Luciferase analysis indicated that GNG13, DR1 and BTG3 are the respectively potential target genes of miR-513a, miR-513b and miR-513c. Previously, Zhang *et al.* reported that the miR-513 subfamily members are preferentially expressed in testis, and that expression decreases sharply during the course of sexual maturation in male rhesus macaques, suggesting that miR-513 may play some unknown functional roles in testis [6]. Based on this finding, we tested the expression of GNG13, DR1 and BTG3 in testis tissues of both infant and adult male rhesus macaques. In both infant and adult rhesus testis, DR1 and BTG3 are expressed, but GNG13 is undetectable. Interestingly, a significantly increased expression of DR1 and BTG3 in testis of adult macaques was observed as compared with the infant macaques measured by real-time quantitative PCR (Figure 5), which is negatively correlated with the expression of the miR-513 members [6]. Our observation suggests that miR-513b and miR-513c may affect the development of testis by regulating DR1 and BTG3.

### MiR-513b down-regulates the expression of DR1 at mRNA and protein levels

**Figure 6:**
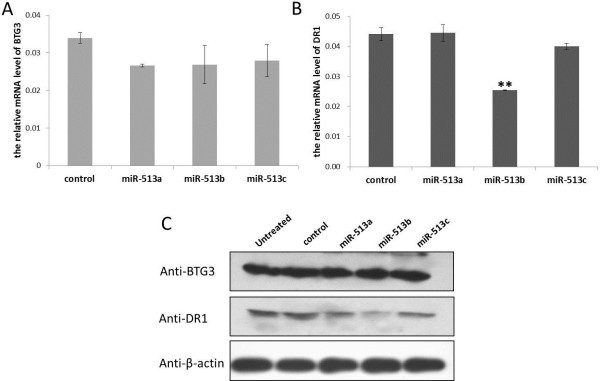
**Significantly decreased expression of DR1 both at the mRNA and protein levels by MiR-513b. (A)** mRNA levels of BTG3 in transfected HeLa cells by real-time qPCR. **(B)** mRNA levels of DR1 in transfected HeLa cells by real-time qPCR. **(C)** protein levels of BTG3 and DR1 in transfected HeLa cells via western blotting. β-actin was used as the internal control gene. “untreated” represents untransfected cells. “control” represents cells transfected by negative control miRNA. Error bar represents standard deviation (n = 3). ** *P* < 0.01 (two-tailed student’s t-test).

To further demonstrate whether miR-513b and miR-513c can inhibit the expression of DR1 and BTG3, we measured both mRNA and protein expression levels of endogenous DR1 and BTG3 in HeLa cells, after introducing the synthetic miR-513a/b/c and the negative control miRNAs. We found that miR-513b could significantly decrease the expression of DR1 both at the mRNA and protein levels, while miR-513c had no obvious impact on the mRNA and protein expression level of BTG3 (Figure 6).

## Discussion

MiR-513 is an X-linked miRNA subfamily with multiple copies that has undergone rapid evolution in primates. Sequence comparisons revealed that the duplicated copies of miR-513 were derived from transposable elements (MER91C), different from other members within the miR-506-514 cluster. Zhang *et al.* showed that the duplications of another subfamily (miR-514) in this cluster occurred independently in humans and chimpanzees [6]. By contrast, the initial duplication event of the miR-513 subfamily seems to have occurred earlier in the common ancestor of *Catarrhini.* Together, these results indicated that the miR-513 subfamily has a unique evolutionary history, quite different from other members in the same miRNA cluster.

Different copies of the miR-513 subfamily (miR-513a/b/c) with diverged seed sequences seem to have led to functional divergences. Our results showed that miR-513a, miR-513b and miR-513c are capable of specifically targeting their predicted sites in the 3′UTRs of GNG13, DR1 and BTG3 respectively. GNG13 is a guanine nucleotide binding protein subunit, expressed in taste, retinal, and neuronal tissues, and earlier studies reported GNG13 playing a key role in taste transduction [8]. In contrast to one or several miRNA binding sites in the 3′ UTRs of target genes, there are 13 target sites of miR-513a in the 3′ UTR of GNG13, because the 3′ UTR of GNG13 contains 13 repeat elements matching the recognition site of miR-513a, resulting in a strong miR-513a-dependent inhibition of luciferase expression. Additionally, Gong *et al.* reported that miR-513a could regulate B7-H1’s translation and is involved in the IFN-gamma signal pathway in cholangiocyte [9]. These findings suggest that the functional role of miR-513a is likely diverged from miR-513b/c and may not be related to male reproduction—an implication of neo-functionalization.

DR1 (down-regulator of transcription 1) is a TBP (TATA box-binding protein) associated phosphoprotein that regulates both basal and activated levels of transcription [10]. BTG3 is a member of the BTG3/Tob family that has antiproliferative properties [11]. Previous reports offered no substantive proof as to whether DR1 or BTG3 is involved in testicular development. However, over the course of our study we observed significant up-regulation of both DR1 and BTG3 in the testis after sexual maturation of male rhesus macaques, and a negative correlation between the expression of DR1/BTG3 and miR-513, suggesting miR-513′s role on testicular development via its regulation of DR1 and BTG3. Moreover, we showed that miR-513b could inhibit the expression of DR1 *in vitro* at both the mRNA and protein levels. TBP (a DR1 associated protein) has been reported to play an important role on spermatogenesis [12]. Taken together, these findings prompt us to propose that miR-513b affects male sexual maturation by negatively regulating the development-stage-related functioning of DR1. Although there is no solid functional evidence, miR-513c emerged only in apes and humans, but shown sequence divergence in different lineages, implying that it may contribute to lineage-specific functions.

## Conclusions

In summary, we demonstrated that the miR-513 subfamily underwent multiple independent gene duplications among different lineages of primates. More importantly, the after-duplication sequence divergences among the copies of miR-513 have led to functional divergence of these copies in primates.

## Methods

### Sequence analysis and target gene prediction

MiR-513 precursor and mature sequences were retrieved from the miRBase database (http://www.mirbase.org) and remapped to reference genomes of four primate species in UCSC using BLAT with default setting, including gorilla (gorGor3), orangutan (ponAbe2), gibbon (nomLeu3), and mouse lemur (micMur1). The genomic locations and sequences of the miR513s were compared to transposable elements (TEs) annotated with the RepeatMasker program (http://www.repeatmasker.org/). The criteria were ≥50% sequence identity and ≥80% location overlap. For phylogenetic analysis, precursor sequences were aligned by ClustalW (Gap opening 15; Gap extension 6.66; Transition Weight: 0.5) [13]. Phylogenetic trees were reconstructed using the neighbor-joining method and maximum likelihood in MEGA5 and evaluated with 1,000 bootstrap replications [14]. AnGST was also used to construct the gene tree by merging an ensemble of trees from different bootstrapping into a single “chimeric” tree (http://almlab.mit.edu/angst/). Tree ensemble used for AnGST was generated by the non-parametric bootstrapping step of PhyML (1,000 bootstrap replications) (http://www.atgc-montpellier.fr/phyml/). In order to infer gene duplication and loss events, gene tree and species tree reconciliation was performed by AnGST and Notung (http://www.cs.cmu.edu/~durand/Notung/). The species tree was based on the NCBI taxonomy tree (http://www.ncbi.nlm.nih.gov/taxonomy). To identify potential targets of miR-513 sequences, we used TargetScanS [15], and eight genes with top rank values were selected for functional testing.

### DNA constructs

All the constructs for this study were derived from psiCHECK™-2 vector (Promega, C8021). Human 3′UTR sequences were amplified from genomic DNA of human white blood cells using PCR. The oligonucleotide primers used for the generation of constructs are shown in Additional file 1: Table S2. PCR products were sub-cloned into psiCHECK™-2 vector. All constructs were then verified by sequencing. All synthesized miR-513a/b/c mimics (miR10002877, miR1000578 and miR10005789) and the mimic negative control (miR01201) were purchased from RiboBio (China).

### Cell culture, transfection and tissue preparation

HeLa and HEK293T cells were grown in high glucose DMEM containing 10% FBS (HyClone, Logan, USA). They were transiently transfected with DNA constructs and miRNA mimics at 40% confluence in 6 or 24 well plates using Lipofectamine 2000 (Invitrogen). Cells were lysed 24–72 hours after transfection for luciferase assay, real time PCR, or western blotting. Three infant (~2 years old) and three adult (~8 years old) rhesus macaque testis samples were used in this study.

### Luciferase assay

After transiently transfected with DNA constructs and miRNA mimics, HeLa or HEK293T cells are lysed by Reporter Lysis Buffer for 10–20 min. The luciferase activity in cell extracts was determined by Dual-luciferase Reporter Assay System (Promega, E1910) according to the manufacturer’s protocols. The relative light units were measured using a luminometer.

### Real time quantitative PCR

Total RNA was isolated from tissues or transfected cells. These RNAs were reverse-transcribed with oligo-dT (20) primer and amplified by real time primers. Real time quantitative PCR (qPCR) reactions (15 μl total volume containing 0.5 μl 10 μM primer, 7.5 μl SYBR Green dye (Bio-Rad, CA, USA), and 2 μl of cDNA) were carried out with a DNA Engine Opticon Continuous Fluorescence Detection System (MJ Research Inc, Waltham, MA) for 40 cycles. The Ct values for each gene amplification were normalized by subtracting the Ct value calculated for ATCB. Normalized gene expression values were taken as the relative quantity of BTG3 or DR1 gene-specific messenger RNA (mRNA). The oligonucleotide primers used in the qPCR amplifications are shown in Additional file 1: Table S3.

### Western blotting

Protein from transfected cells was homogenized in RIPA buffer (Beyotime, P0013) containing a cocktail of protease inhibitor (Sigma Chemical, MO, USA). Extracted protein (15–20 μg) was separated by SDS-polyacrylamide gel electrophoresis and transferred to a membrane by electrophoretic transfer. The membrane was incubated withβ-actin monoclonal antibody (Abcam, ab6276, 1:5000), anti-BTG3 polyclonal antibody (Abcam, ab112938, 1:500) and anti-DR1 polyclonal antibody (Abcam, ab88597,1:500). Then the membrane was incubated with HRP-conjugated secondary antibodies. Immunoreactivity was detected with an enhanced chemiluminescence system (Pierce, IL, USA) with colored markers (Fermentas) as molecular size standards.

## Availability of supporting data

Phylogenetic data used in this study were submitted to TreeBASE (http://purl.org/phylo/treebase/phylows/study/TB2:S14738?x-access-code=9db8bc2ae28ac495564c94409d1023e8&format=html).

## Competing interests

The authors declare that no competing interests.

## Authors’ contributions

ZS: data collection, assembly, analysis, and manuscript writing; YZ, RZ and XQ: data collection and analysis; BS: conception and design, data interpretation, financial support, writing and approving the final manuscript. All authors read and approved the final manuscript.

## Supplementary Material

Additional file 1: Table S1 MiR-513 precursor sequences without annotation in miRbase. **Table S2.** The miR-513 copies co-localized with MER91C and their sequence similarity. **Table S3.** PCR primers were used in this study. **Figure S1.** The ML tree of the miR-513 subfamily. **Figure S2.** The chimeric gene tree of the miR-513 subfamily constructed by AnGST. **Figure S3.** The reconciliation tree that combining gene tree and species tree. **Figure S4.** Alignment of the miR-513 precursor sequences. **Figure S5.** Alignments between miR-513a/b/c and their binding sites of the target genes. **Figure S6.** Other five candidate target genes tested in the luciferase assay. Click here for file
