# Supplementary material for: Functional divergence of the rapidly evolving miR-513 subfamily in primates
Source: BMC Evol Biol. 2013 Nov 19;13:255. doi: 10.1186/1471-2148-13-255 (PMC3840687; doi:10.1186/1471-2148-13-255)
Supplement: Additional file 1: Table S1 — MiR-513 precursor sequences without annotation in miRbase. Table S2. The miR-513 copies co-localized with MER91C and their sequence similarity. Table S3. PCR primers were used in this study. Figure S1. The ML tree of the miR-513 subfamily. Figure S2. The chimeric gene tree of the miR-513 subfamily constructed by AnGST. Figure S3. The reconciliation tree that combining gene tree and species tree. Figure S4. Alignment of the miR-513 precursor sequences. Figure S5. Alignments between miR-513a/b/c and their binding sites of the target genes. Figure S6. Other five candidate target genes tested in the luciferase assay. [file 1471-2148-13-255-S1.doc]

**Table S1.** MiR-513 precursor sequences without annotation in miRbase.

| >pan-mir-513-b |
| --- |
| TGTACAGTGCCTTTCACAAGGAGGTGTCATTTATGTGAACTAAACTATAAATGTCACCTTTtTGGGAAGAGTAATGTACA |
| >pan-mir-513-a1 |
| TGTACAGTGCCTTTCACAGGGAGGTGTCATTTATGTGAACTAAAATATAAATTTCACCTTTCTGAGAAGAGTAATGTACA |
| >pan-mir-513-a2 |
| TATACAGTGCCTTTCACAGGGAGGTGTCATTTATGTGAACTAAACTATAAATGTCACCTTTCTGCGAAGGGTAATGTACA |
| >pan-mir-513-a3 |
| TGTACAGTGCCTTTCACAGGGAGGTGTCATTTATGTGAACTAAAATATAAATTTCACCTTTCTGAGAAGAGTAATGTACA |
| >pan-mir-513-a4 |
| TGTACAGTGCCTTTCACAGGGAGGTGTCATTTATGTGAACTAAACTATAAATGTCACTTTTCTGAGAAGAGTAATGTACA |
| >pha-mir-513c |
| TGTACAGTGCCTTTCCCAAGGAGGTGTCATTTATGTGAACTAAAATATAAATTTCACCTTTCTGAGAAGAGTAATGTACA |
| >pha-mir-513b1 |
| TGTACAGTGCCTTTCACAAGGAGGTGTCATTTATGTGAACTAAAATATAAATGTGACGTTCTTGAGAAGAGTAATGTACA |
| >pha-mir-513a |
| TGTGCAGTGCCTTTCACAGGGAGGTGTCATTTATGTGAACTAAAATATAAATTTCACCTTTCTGAGAAGAGTAATGTACA |
| >pha-mir-513b2 |
| TGTGCAGTGCCTTTCACAAGGAGGTGTCATTTATGTGAACTAAAATATAAATTTCACCTTTTTGAGAAGGGTAATGTACA |
| >ggo-mir-513c1 |
| TGTACAGTGCCTTTCTCAaGGAGGTGTCATTTATGTGAACTAAAATATAAATTTCACCTTTCTGAGAAGAGTAATGTACA |
| >ggo-mir-513c2 |
| TGTACAGTGCCTTTCTCAAGGAGGTGTCATTTATGTGAACTAAAATATAAATTTCACCTTTCTGAGAAGAGTAATGTACA |
| >ggo-mir-513b |
| TGTACAGTGCCTTTCACAAGGAGGTGTCATTTATGTGAACTAAAATATAAATGTCACCTTTTTGAGAGGAGTAATGTACA |
| >ggo-mir-513a |
| TGTACAATGCCTTTCACAGGGAGGTGTCATTTATGTGAACTAAAATATAAATTTCACCTTTCTGAGAAGGGTAATGTACA |
| >lca-mir-513bL |
| TGTGCAGTGCGTTTCACAAGAAGGTGTCATTCATGTGAGCCAAAATATGAATGGCACATTTTTGAGAAATGTAATGTACA |

**Table S2.** The miR-513 copies co-localized with MER91C and their sequence

similarity.

| **Name** | **TE** | **Overlap** | **Divergence** | **Deletions** | **Insertions** | **Identify** |
| --- | --- | --- | --- | --- | --- | --- |
| has-mir-513a1 | MER91C | 100% | 40.5% | 5.3% | 0 | 54.2% |
| has-mir-513a2 | MER91C | 100% | 39.2% | 5.4% | 0 | 55.4% |
| has-mir-513b | MER91C | 100% | 37.7% | 5.4% | 0 | 56.9% |
| has-mir-513c | MER91C | 100% | 40.0% | 5.4% | 0 | 54.6% |
| ptr-mir-513a1 | MER91C | 100% | 40.0% | 5.4% | 0 | 54.6% |
| ptr-mir-513a2 | MER91C | 100% | 40.0% | 5.4% | 0 | 54.6% |
| ptr-mir-513a3 | MER91C | 100% | 39.2% | 5.4% | 0 | 55.4% |
| ptr-mir-513b | MER91C | 100% | 38.5% | 5.4% | 0 | 56.1% |
| ggo-mir-513a | MER91C | 100% | 40.0% | 5.3% | 0 | 54.7% |
| ggo-mir-513b | MER91C | 100% | 37.7% | 5.3% | 0 | 57.0% |
| ggo-mir-513c1 | MER91C | 100% | 38.55% | 5.3% | 0 | 56.2% |
| ggo-mir-513c2 | MER91C | 100% | 38.5% | 5.3% | 0 | 56.2% |
| ppy-mir-513a1 | MER91C | 100% | 40.0% | 5.3% | 0 | 54.7% |
| ppy-mir-513a2 | MER91C | 100% | 38.5% | 5.3% | 0 | 56.2% |
| ppy-mir-513aL | MER91C | 100% | 40.0% | 5.3% | 0 | 54.7% |
| ppy-mir-513b | MER91C | 100% | 36.9% | 5.3% | 0 | 57.8% |
| pha-mir-513a | MER91C | 100% | 38.3% | 5.3% | 0 | 56.4% |
| pha-mir-513b1 | MER91C | 100% | 40.0% | 5.3% | 0 | 54.7% |
| pha-mir-513b2 | MER91C | 100% | 37.4% | 5.3% | 0 | 57.3% |
| pha-mir-513c | MER91C | 100% | 39.7% | 5.3% | 0 | 55.0% |
| ssy-mir-513a | MER91C | 100% | 40.5% | 5.3% | 0 | 54.2% |
| ssy-mir-513b2 | MER91C | 100% | 37.4% | 5.3% | 0 | 57.3% |
| mml-mir-513a2 | MER91C | 100% | 39.7% | 5.3% | 0 | 55.0% |
| mml-mir-513b1 | MER91C | 100% | 34.9% | 9.2% | 3.8% | 52.1% |
| mml-mir-513b2 | MER91C | 100% | 37.4% | 5.3% | 0 | 57.3% |
| pan-mir-513a1 | MER91C | 100% | 39.7% | 5.3% | 0 | 55.0% |
| pan-mir-513a3 | MER91C | 100% | 39.7% | 5.3% | 0 | 55.0% |
| pan-mir-513b | MER91C | 100% | 39.7% | 5.3% | 0 | 55.0% |
| pbi-mir-513b | MER91C | 100% | 35.7% | 6.5% | 1.5% | 56.3% |
| age-mir-513bL1 | MER91C | 100% | 34.1% | 6.9% | 1.5% | 57.5% |
| age-mir-513bL2 | MER91C | 100% | 35.0% | 7.5% | 0 | 57.5% |
| age-mir-513bL3 | MER91C | 100% | 35.9% | 5.3% | 0 | 58.8% |
| age-mir-513cL1 | MER91C | 100% | 36.1% | 6.2% | 0 | 57.7% |
| age-mir-513cL2 | MER91C | 100% | 35.1% | 5.3% | 0 | 59.6% |
| age-mir-513d | MER91C | 100% | 35.7% | 9.2% | 3.8% | 51.3% |
| age-mir-513e1 | MER91C | 100% | 34.4% | 7.0% | 0.8% | 57.8% |
| age-mir-513e2 | MER91C | 100% | 33.1% | 8.5% | 2.3% | 56.1% |
| lca-mir-513bL | MER91C | 100% | 35.1% | 5.3% | 0 | 59.6% |

**Table S3.** PCR primers were used in this study.

| **Primer ID** | **Sequence** |
| --- | --- |
| BTG3-RT-F | CGCAAGTCCTGTGTACCAGAT |
| BTG3-RT-R | GCCATTCCCTCGATACATTCCT |
| BTG3-1140-xho1 | CCGCTCGAGGCACCTCACTAACTTCGTTTTTG |
| BTG3-1566-not1 | ATTGCGGCCGCGCAACTACATGATTTCACACAATTC |
| CTNS-h24625-xho1 | CCGCTCGAGTCCACCTTCTCAGATTAGCC |
| CTNS-h26641-not1 | ATTGCGGCCGCGCCTGTGGGTTTAACTTTTG |
| DR1-h14807-xho1 | CCGCTCGAGTATGCATCTTGGTGGACTTG |
| DR1-h16864-not1 | ATTGCGGCCGCATGCCTCGATCTTAGGACTG |
| DR1-RT-F | AGAAAGGCCAGTTCTCGTTTG |
| DR1-RT-R | GCTTGTTGCTGTCTAGCTTTTG |
| GNG13-RT30 | TCATTGTCCCTCCGCTGTC |
| GNG13-RT184 | TGGTCTTGGACGCCATCTC |
| GNG13-h2949s-xho1 | CCGCTCGAGCTTCCTGAACCCCGACCTGATG |
| GNG13-h3715a-not1 | AGCGGCCGCTGCAGAGACAGGAAGCTGGAGATG |
| IL13RA1-1296-pme1 | CCGTTTAAACTGATAGAAAACCTGAAGAAAGCCTC |
| IL13RA1-3287-not1 | ATTGCGGCCGCGCACCATGCTTCTACACGGC |
| NIPAL4-1531-xho1 | CCGCTCGAGTGAGTGAGAGGATGAGTCCGATG |
| NIPAL4-3253-not1 | ATTGCGGCCGCATTCTTGACCCTGCAGACCACAC |
| psi1611 | GCTGAAGAACGAGCAGTAAT |
| psi1726 | GCCAACACACAGATGTAATG |
| PI4K2B-1759-xho1 | CCGCTCGAGTTCAGGGAAGAAGTGCTATATCTC |
| PI4K2B-3458-not1 | ATTGCGGCCGCCCTTTAGTCTACCACATTTGTCAC |
| YBX2-h1197-xho1 | CCGCTCGAGTGCCAGTTTTTCCAAATGACC |
| YBX2-h1549-not1 | ATTGCGGCCGCGCTCAGGTGGCACATGACAGA |

**
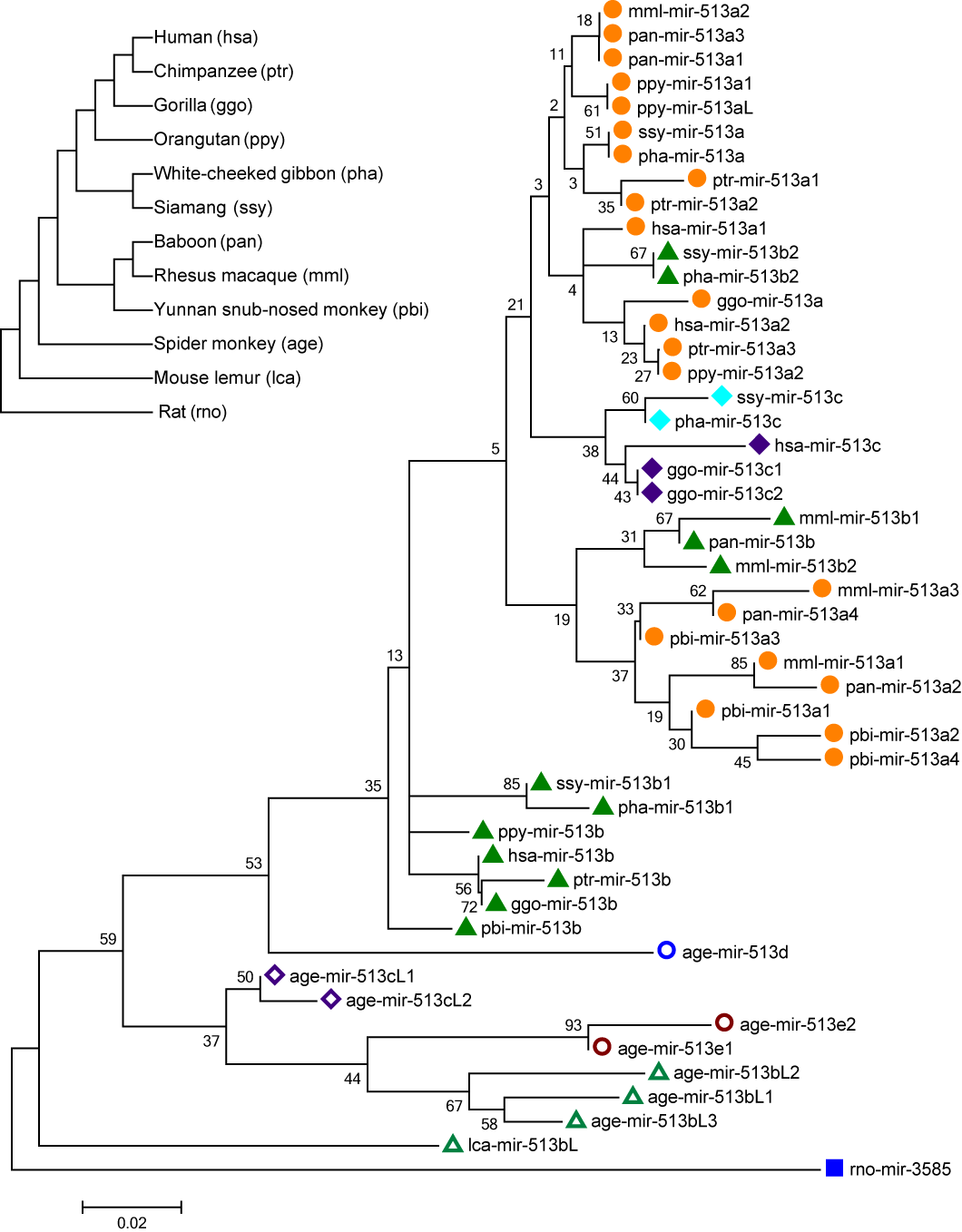
**

**Figure S1. The ML tree of the miR-513 subfamily.** The tree was reconstructed using the maximum likelihood method in MEGA5 with 1,000 bootstrap replications (bootstrap values are labeled along the branches). Copies marked with the same colors and shapes have the same or similar mature miRNA sequences. The left top tree showed the species tree and the abbreviations for miRNA nomenclature. The scale bar reflects 0.02-nt substitution per site.

**
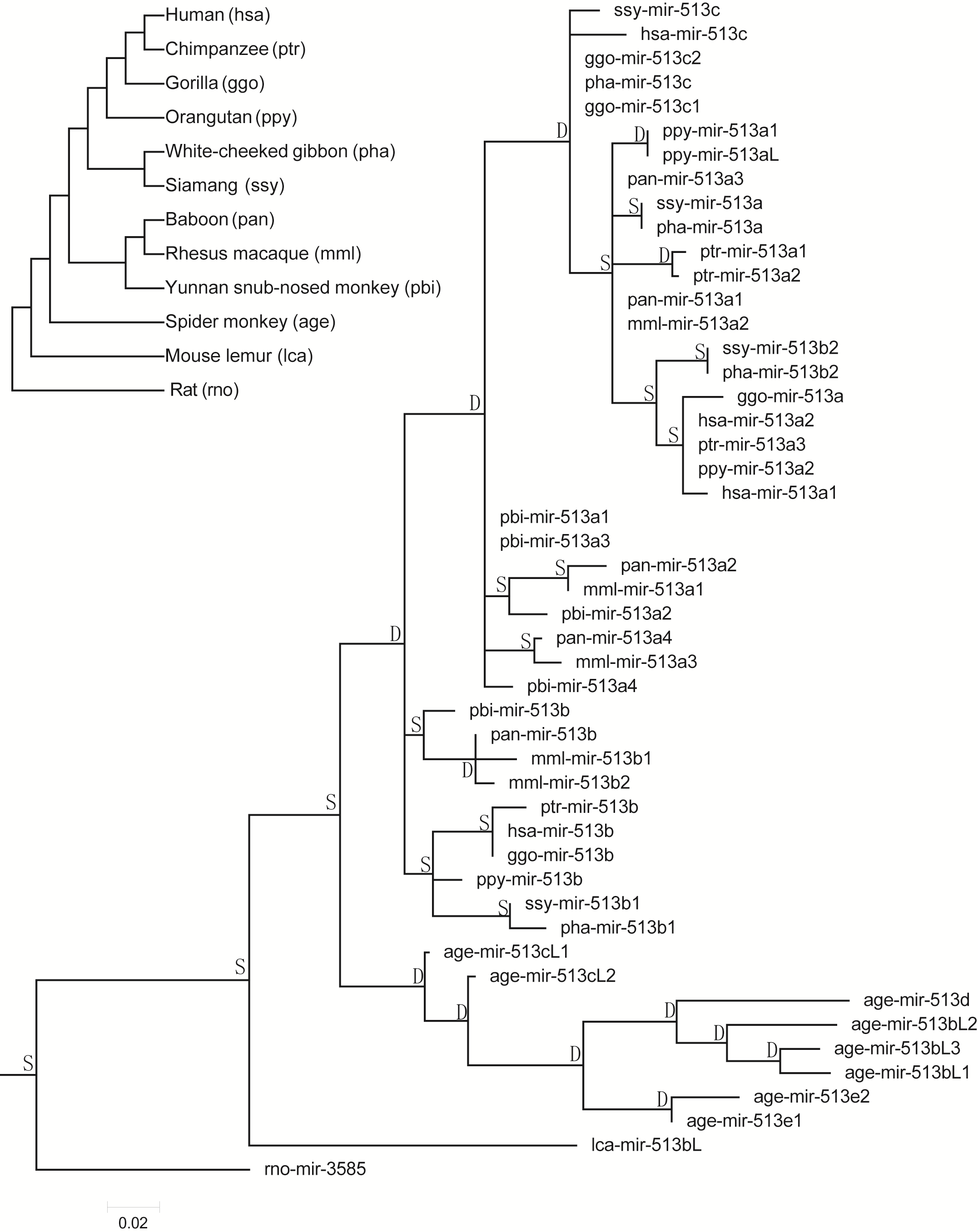
**

**Figure S2.** **The chimeric gene tree of the miR-513 subfamily constructed by AnGST.** The tree was reconstructed using AnGST from an ensemble of trees generated by the non-parametric bootstrapping step of PhyML (1,000 bootstrap replications). The speciation (“S”) and duplication (“D”) events are labeled on the tree. The left top tree showed the species tree and the abbreviations for miRNA nomenclature. The scale bar reflects 0.02-nt substitution per site.

**
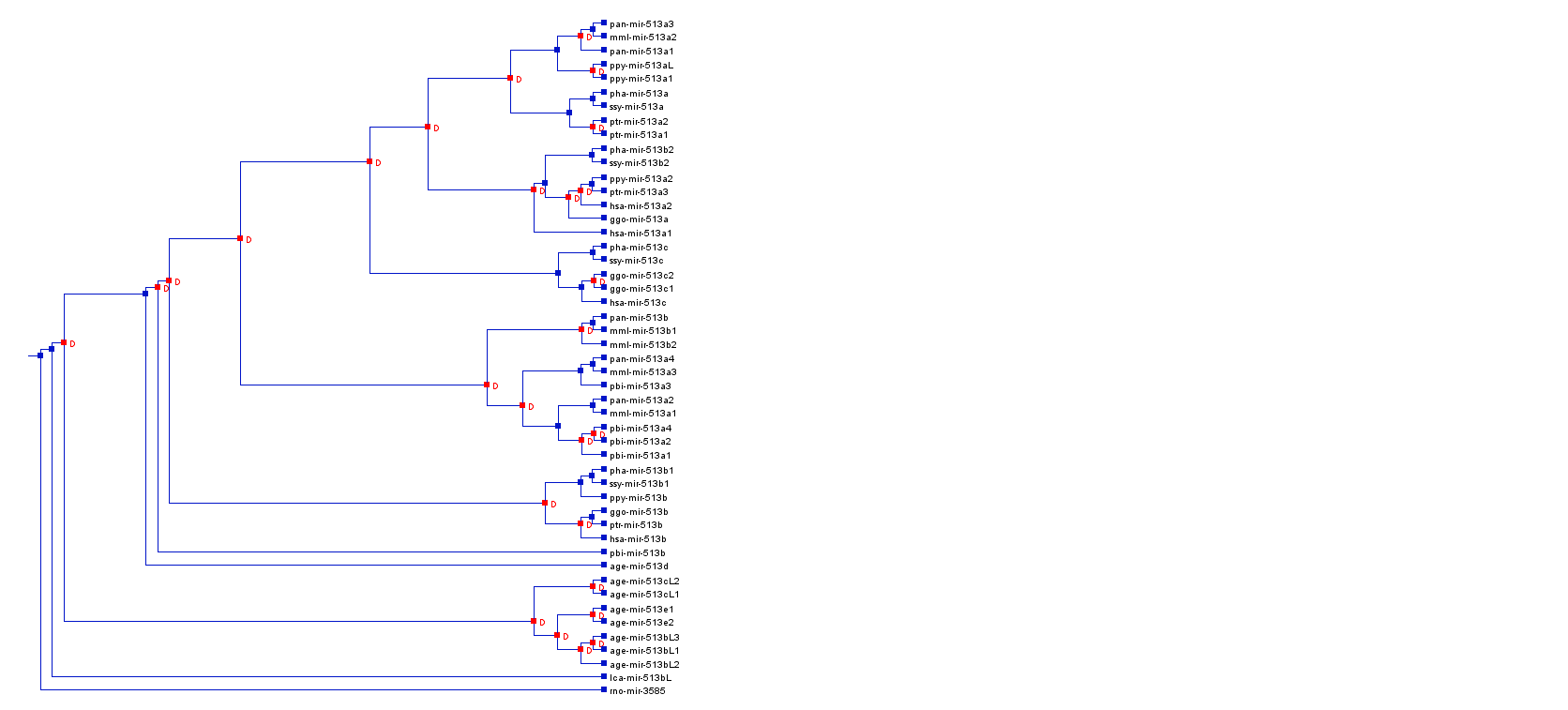
**

**Figure S3.** **The reconciliation tree that combining gene tree and species tree.**The NJ tree was used as the gene tree, and the duplication events (“D”) are labeled on the tree.

**Figure S4.** **Alignment of the miR-513 precursor sequences.** “.” represents consensus sequence and “—” indicates deletion.

**
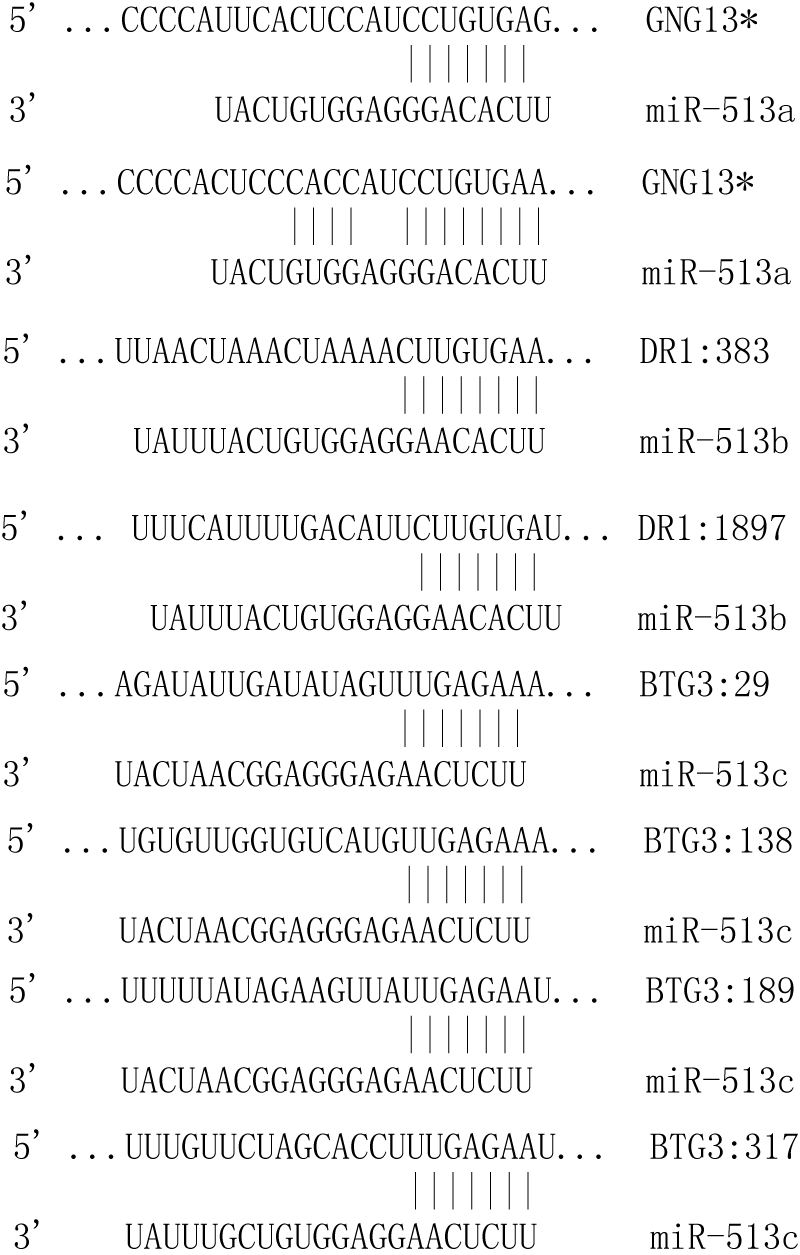
**

**Figure S5. Alignments between miR-513a/b/c and their binding sites of the target genes.** Numbers represent the start position in the 3’UTR of target genes. “*” represent multiple positions in GNG13 3’UTR containing the same binding sites.


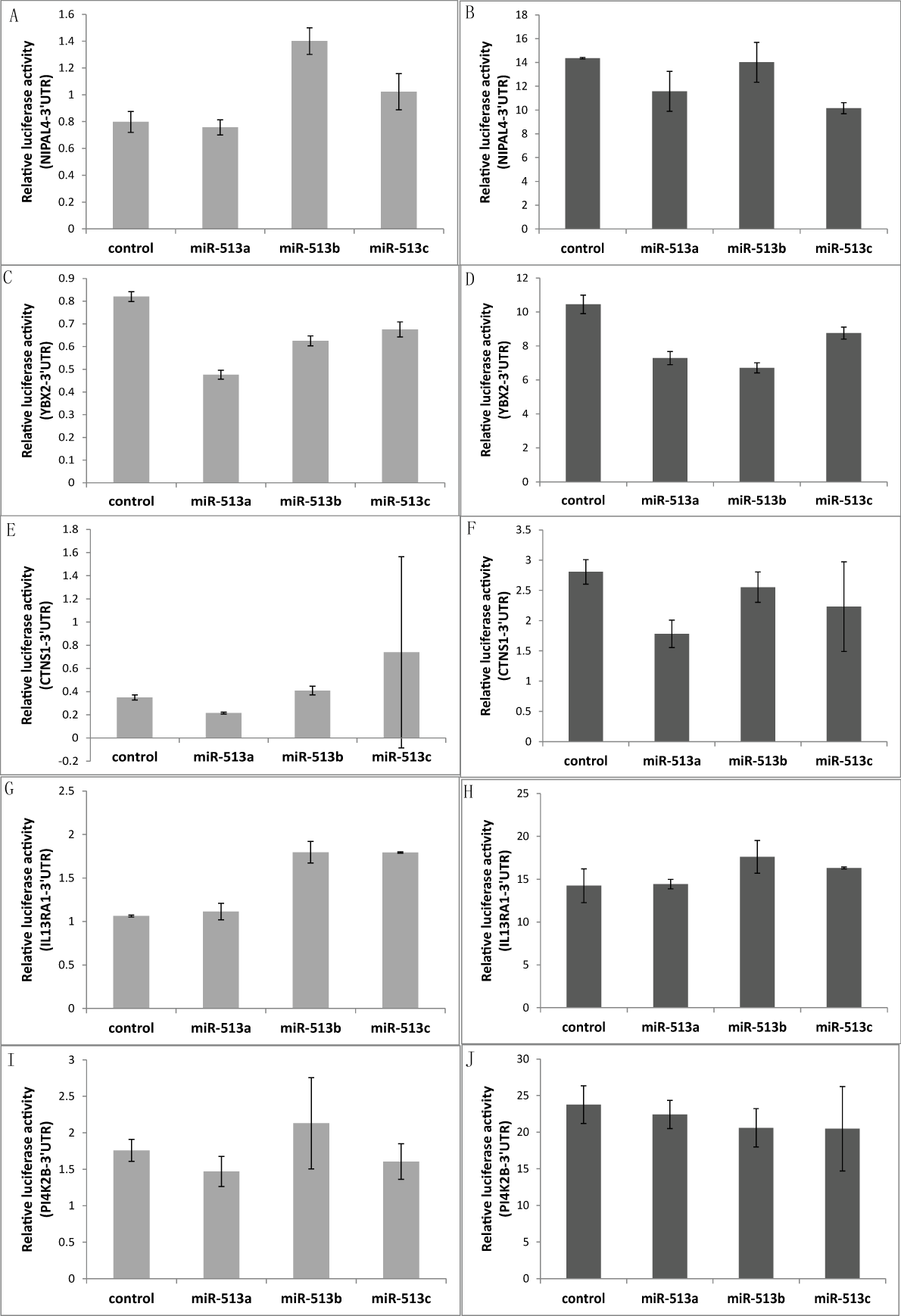


**Figure S6. Other five candidate target genes tested in the luciferase assay.** Value on Y axis represents the ratio of Renilla luciferase (with candidate target gene’s 3’ UTR) activity to firefly luciferase activity after treated with control or miR-513 mimics. A, C, E, G, I are results in HEK293T cells, while B, D, F, H, J are results in HeLa cells. Error bars represent standard deviations (n=3). ** P< 0.01 (two-tailed student’s t-test).
